# Enolase 1 Correlated With Cancer Progression and Immune-Infiltrating in Multiple Cancer Types: A Pan-Cancer Analysis

**DOI:** 10.3389/fonc.2020.593706

**Published:** 2021-02-10

**Authors:** Wenhua Xu, Wenna Yang, Chunfeng Wu, Xiaocong Ma, Haoyu Li, Jinghui Zheng

**Affiliations:** ^1^ Department of Cardiology, Ruikang Hospital Affiliated to Guangxi University of Chinese Medicine, Nanning, China; ^2^ Graduate School, Guangxi University of Chinese Medicine, Nanning City, China; ^3^ Department of Ophthalmology, Jingliang Eye Hospital Affiliated to Guangxi Medical University, The First Affiliated Hospital of Guangxi University of Chinese Medicine, Nanning, China

**Keywords:** Enolase 1 (ENO1), biologic tumor marker, mutation, tumor-infiltrating, prognosis

## Abstract

Enolase 1 (ENO1) is an oxidative stress protein expressed in endothelial cells. This study aimed to investigate the correlation of ENO1 with prognosis, tumor stage, and levels of tumor-infiltrating immune cells in multiple cancers. ENO1 expression and its influence on tumor stage and clinical prognosis were analyzed by UCSC Xena browser, Gene Expression Profiling Interactive Analysis (GEPIA), The Cancer Genome Atlas (TCGA), and GTEx Portal. The ENO1 mutation analysis was performed by cBio Portal, and demonstrated ENO1 mutation (1.8%) did not impact on tumor prognosis. The relationship between ENO1 expression and tumor immunity was analyzed by Tumor Immune Estimation Resource (TIMER) and GEPIA. The potential functions of ENO1 in pathways were investigated by Gene Set Enrichment Analysis. ENO1 expression was significantly different in tumor and corresponding normal tissues. ENO1 expression in multiple tumor tissues correlated with prognosis and stage. ENO1 showed correlation with immune infiltrates including B cells, CD8^+^ and CD4^+^ T cells, macrophages, neutrophils, and dendritic cells, and tumor purity. ENO1 was proved to be involved in DNA replication, cell cycle, apoptosis, glycolysis process, and other processes. These findings indicate that ENO1 is a potential prognostic biomarker that correlates with cancer progression immune infiltration.

## Introduction

Enolase 1 (ENO1) is an oxidative stress protein expressed in endothelial cells. It plays an essential role in the glycolytic pathway by converting 2-phosphoglycerate to phosphoenolpyruvate (1) and functions as a critical contributor to Warburg effect in cancer cells (2). Recent evidence shows that some enzymes responsible for glycolysis are complicated, multifaceted proteins rather than simple components of the glycolytic pathway (3, 4). The energy produced by glycolysis is used not only for tumor growth but also for tumor tolerance, such as the discharge of anticancer drugs and their metabolites from cancer cells (5). ENO1 is involved in a series of physiological processes, such as autoimmunity, hypoxia tolerance, and cell growth (6, 7). In particular, ENO1 expressed on the cell surface has been shown to promote migration and metastasis of tumor cells by inducing plasminogen activation and extracellular matrix degradation as a plasminogen receptor (8).

Besides its major role in glycolysis, ENO1 is also considered as a multifunctional protein demonstrating various distinct activities (9). Previous studies found that the upregulation of ENO1 was positively correlated with progression and poor prognosis in breast cancer, prostate cancer, thyroid carcinoma, hepatocellular carcinoma, cholangiocarcinoma, neuroblastoma, neuroendocrine tumors, lung cancer, and pancreatic cancer (4, 9–13). Consistent to previous studies (2, 4, 14–23), more details are summarized in **Supplementary Table 1**. Additionally, increased ENO1 expression has been observed in different types of drug-resistant cancer cells, suggesting the potential use of ENO1 as a biomarker for drug resistance and as a target for cancer therapy (5). ENO1 also involves in cell adhesion-mediated resistance in non-Hodgkin lymphoma and tamoxifen resistance in breast cancer (24). ENO1 has been shown to induce autoantibodies in patients with cholangiocarcinoma, breast cancer, head and neck cancer, leukemia, lung cancer, pancreatic cancer and melanoma (25–28). The correlation between tumor and autoimmunity may be due to the immunogenicity and proinflammatory stimulation produced by tumor cell death, as well as the activation of inflammatory process in tumor microenvironment, thus increasing the expression of autoantigen to the immune system (29). ENO1 is a major auto-antigen. ENO1 specific T cells from peripheral blood to tumor are inhibited by a number of immunosuppressive mechanisms (17). Their presence in the peripheral blood is associated with the prevention of metastasis by excision of cancer circulating cells (30, 31). One explanation may be that tumor cells physically absorb and neutralize ENO1 antibodies expressed and secreted on the surface to reduce circulating levels.

The recently completed Cancer Genome Atlas (TCGA) project provides matched clinical and molecular data of multiple cancers, which facilitates systematical analysis of the survival impact of single gene expression. The correlation of ENO1 expression with prognosis, tumor stage, and levels of tumor immune infiltrates in different cancers remains unclear.

In this study, we performed a pan-cancer analysis of tumor and normal samples from TCGA dataset to evaluate the impacts of ENO1 on prognosis, staging, and immune infiltrating levels in 23 cancer types, including cervical squamous cell carcinoma and endocervical adenocarcinoma (CESC), lung adenocarcinoma (LUAD), and kidney chromophobe (KICH).

## Materials and Methods

### Genomic Data Collection

The clinical information and expression levels of ENO1 in 33 types of cancers were obtained from UCSC Xena browser (https://xena.ucsc.edu/; accessed by April 20, 2020) (32) and The Cancer Genome Atlas (TCGA, https://portal.gdc.cancer.gov/; accessed by April 20, 2020).

### ENO1 Gene Expression Analysis

The expression levels of ENO1 in normal tissues were identified in GTEx Portal (https://www.gtexportal.org/home/; accessed by April 20, 2020). The boxplots of ENO1 expression in different types of cancers were constructed by Gene Expression Profiling Interactive Analysis (GEPIA, http://gepia.cancer-pku.cn/index.html; accessed by April 20, 2020) (33).

### Correlation of ENO1 Expression to Prognosis and Tumor Stage

To evaluate the prognostic potential of ENO1 in cancers, the correlations between ENO1 expression and survival outcomes of cancer patients, including OS and disease-free survival (DFS), were investigated. Patients were divided into high- and low-expression groups using the 50^th^ percentile of ENO1 expression level as the cutoff value. The association between ENO1 expression and tumor stage was also investigated. The forest plot was generated using the R program (v3.6.1). In the subsequent immune infiltrate analysis and Gene Set Enrichment Analysis (GSEA), cancers were included if (1) tumor samples showed significant changes in ENO1 expression compared to normal tissues, and (2) a significant correlation between ENO1 expression and prognosis was found.

### Mutation Analysis of ENO1

In this study, the cBio Cancer Genomics Portal (http://cbioportal.org; accessed by April 30, 2020), which is a web tool for mutation analysis and visualization through TCGA cancer genomics profiles (34, 35), was used for mutation analysis of ENO1. The genetic alteration of ENO1 and impact of alteration situation on prognosis in multiple cancer types were analyzed and visualized *via* cBio Portal data.

### Immune Infiltrates

The Tumor Immune Estimation Resource database (TIMER, https://cistrome.shinyapps.io/timer/; accessed by April 20, 2020) includes gene expression profiles of 32 types of cancers from TCGA to estimate the abundance of immune infiltrates (36). The expressions of ENO1 in these cancers were analyzed. The correlations of ENO1 expression with tumor purity and the abundance of immune infiltrates in CD8^+^ T cells, CD4^+^ T cells, B cells, macrophages, neutrophils, and dendritic cells were explored. Lastly, we assessed how ENO1 expression correlated with the expression of particular immune infiltrating cell subset markers.

### GSEA

GSEA (http://software.broadinstitute.org/gsea/index.jsp; accessed by April 30, 2020) was performed to explore the potential mechanisms involved in the effect of risk score on cancer prognosis (37, 38). The enrichment analysis was performed using the Molecular Signatures Database (MSigDB) of c2 (c2.cp.kegg.v6.1.symbols.gmt) and c5 (c5.all.v6.1.symbols.gmt). The enriched gene sets in the GSEA that reached a nominal significance level of *P* < 0.05 were considered significant.

### Statistical Analysis

The expression levels of ENO1 in normal tissues were identified in GTEx Portal, and gene expression data from the GEPIA database through TCGA data were analyzed using the *P*, fold changes (FC) and ranks. Survival curves and violin plots were constructed by GEPIA. The method for ENO1 expression differential analysis between TCGA tumor and normal tissue was one-way ANOVA (|log_2_FC| ≥ 1.00, *P* < 0.01). *P <*0.05 was considered as statistically significant in other tests.

## Results

### The Expression of ENO1 in Different Types of Cancers

TCGA contains genomic, epigenomic, transcriptomic, and proteomic data for a total of 33 different cancer types. Cancer datasets with incomplete information on overall survival (OS), tumor stage evaluation, or ENO1 expression, or without control samples were excluded from the analysis. Ten cancer types were excluded from analysis due to incomplete information or lack of control samples. Eventually, the impacts of ENO1 expression on the following 23 cancer types were analyzed: breast invasive carcinoma (BRCA), bladder urothelial carcinoma (BLCA), colon adenocarcinoma (COAD), cholangiocarcinoma (CHOL), cervical squamous cell carcinoma and endocervical adenocarcinoma (CESC), esophageal carcinoma (ESCA), head and neck squamous cell carcinoma (HNSC), kidney renal papillary cell carcinoma (KIRP), kidney renal clear cell carcinoma (KIRC), kidney chromophobe (KICH), lung squamous cell carcinoma (LUSC), lung adenocarcinoma (LUAD), liver hepatocellular carcinoma (LIHC), prostate adenocarcinoma (PRAD), pheochromocytoma and paraganglioma (PCPG), pancreatic adenocarcinoma (PAAD), rectum adenocarcinoma (READ), stomach adenocarcinoma (STAD), skin cutaneous melanoma (SKCM), thymoma (THYM), thyroid carcinoma (THCA), and uterine corpus endometrial carcinoma (UCEC).

The characteristic information is summarized in **Table 1**.

**Table 1:** Characteristic information of 23 types of cancer included in this study.

Abbreviation	Full name	Number of samples	
		Tumor	Normal
BLCA	bladder urothelial carcinoma	404	19
BRCA	breast invasive carcinoma	1,085	112
CESC	cervical squamous cell carcinoma and endocervical adenocarcinoma	306	3
CHOL	cholangio carcinoma	36	9
COAD	colon adenocarcinoma	275	41
ESCA	esophageal carcinoma	182	13
HNSC	head and neck squamous cell carcinoma	519	44
KICH	kidney chromophobe	66	25
KIRC	kidney renal clear cell carcinoma	523	72
KIRP	kidney renal papillary cell carcinoma	286	32
LIHC	liver hepatocellular carcinoma	369	50
LUAD	lung adenocarcinoma	483	59
LUSC	lung squamous cell carcinoma	486	50
PAAD	pancreatic adenocarcinoma	179	4
PCPG	pheochromocytoma and paraganglioma	182	3
PRAD	prostate adenocarcinoma	492	52
READ	rectum adenocarcinoma	92	10
SARC	sarcoma	262	2
SKCM	skin cutaneous melanoma	461	1
STAD	stomach adenocarcinoma	408	36
THCA	thyroid carcinoma	512	59
THYM	thymoma	118	2
UCEC	uterine corpus endometrial carcinoma	174	13

**Figure 1:**
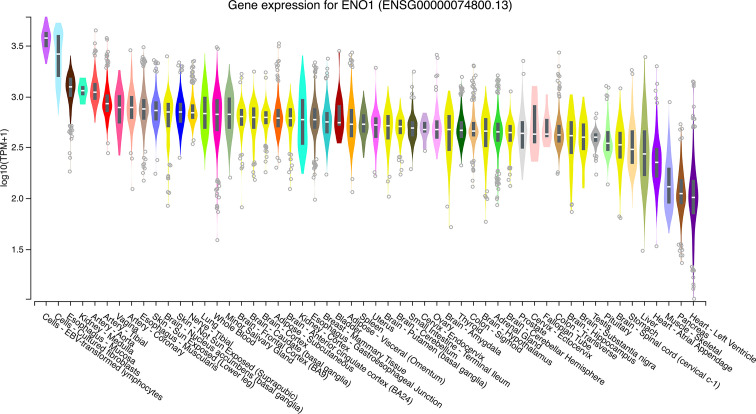
ENO1 expression in normal tissue samples. The expression levels of ENO1 were calculated from a gene model with isoforms collapsed to a single gene and are shown in transcripts per million. The median, 25^th^ and 75^th^ percentiles are shown in the plots. Points that are above or below 1.5 times the interquartile range are shown as outliers. TPM, transcripts per million; ENO1, enolase 1.

**Figure 2:**
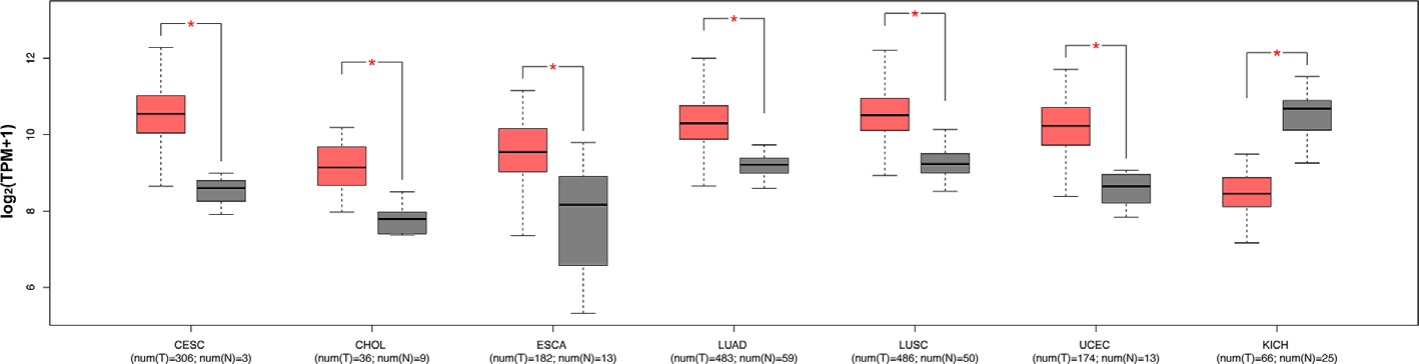
ENO1 expression levels in seven types of cancers with significant differential expression of ENO1 between tumor and normal tissues. The expression levels of ENO1 are shown in transcripts per million. The log_2_FC cutoff and *P* cutoff were set as 1.00 and 0.01, respectively. Pink boxes represent tumor samples and gray boxes represent normal samples. The Y-axes of boxplots represent ENO1 expression in transcripts per million. Plots were generated using GEPIA with data from TCGA. num (T), number of tumor samples; num (N), number of normal samples.

The expression levels of ENO1 in normal tissues are shown in **Figure 1**. The highest ENO1 expression was found in EBV-transformed lymphocytes, whereas the lowest was observed in the left ventricle of the heart. The boxplots of ENO1 expression in tumor and normal tissues were generated by GEPIA (**Figure 2**). The expression levels of ENO1 are shown as TPM. The log_2_FC cutoff and *P* cutoff were set as 1.00 and 0.01, respectively. When compared to the respective normal tissues, ENO1 expression was significantly (*P* < 0.01) elevated in the tissue samples of CESC, CHOL, ESCA, LUAD, LUSC, and UCEC. However, KICH tumor samples showed a significantly lower level of ENO1 compared to normal tissues (*P* < 0.01). The expression of ENO1 in the other 16 cancer types was not significantly different from the respective controls (*P* > 0.01).

### Correlation of ENO1 Expression With Prognosis and Tumor Stage

**Figure 3:**
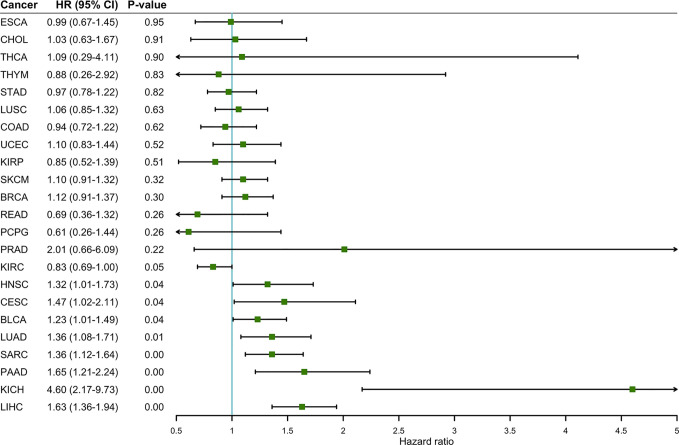
The correlation between ENO1 expression and OS in 23 types of cancers. ENO1 expression significantly impacts prognosis in eight types of cancers, including HNSC, CESC, BLCA, LUAD, SARC, PAAD, KICH, and LIHC. High expression of ENO1 could be considered as an independent risk factor for the above 8 types of cancer (all HR > 1, *P* < 0.05). HNSC, head and neck squamous cell carcinoma; CESC, cervical squamous cell carcinoma and endocervical adenocarcinoma; BLCA, bladder urothelial carcinoma; LUAD, lung adenocarcinoma; SARC, sarcoma; PAAD, pancreatic adenocarcinoma; KICH, kidney chromophobe; LIHC, liver hepatocellular carcinoma; HR, hazard ratio; CI, confidence interval.

**Figure 4:**
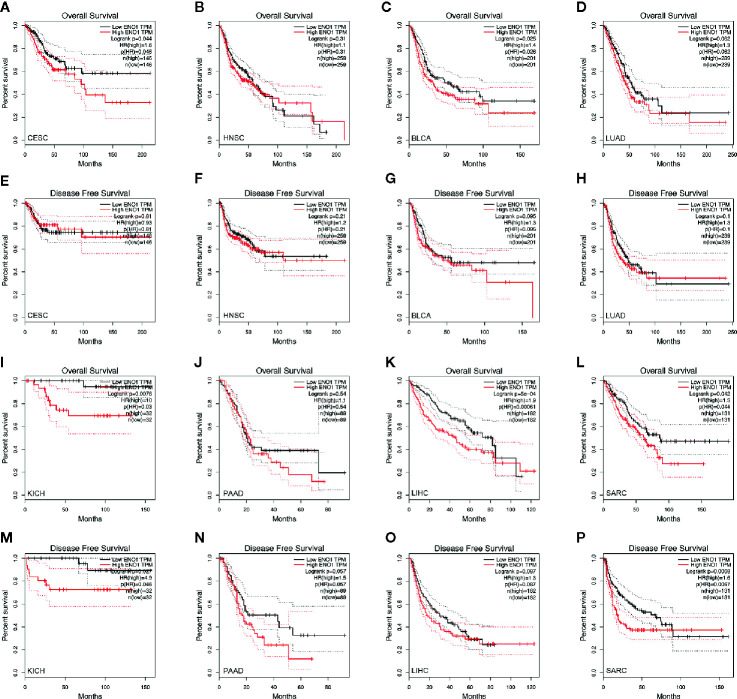
Kaplan–Meier survival curves of the prognostic significance of high- and low-expression of ENO1 in different types of cancers using the GEPIA. Survival curves comparing survival outcomes of patients with high and low expressions of ENO1 in CESC **(A, E)**, HNSC **(B, F)**, BLCA **(C, G)**, LUAD **(D, H)**, KICH **(I, M)**, PAAD **(J, N)**, LIHC **(K, O)** and SARC **(L, P)** with 95% confidence interval. The 1^st^ and 3^rd^ panels show the survival curves for OS, and the 2^nd^ and 4^th^ panels show the survival curves for DFS. ENO1, enolase 1; TPM, transcripts per million; HR, hazard ratio; CESC, cervical squamous cell carcinoma and endocervical adenocarcinoma; HNSC, head and neck squamous cell carcinoma; BLCA, bladder urothelial carcinoma; LUAD, lung adenocarcinoma; KICH, kidney chromophobe; PAAD, pancreatic adenocarcinoma; LIHC, liver hepatocellular carcinoma; SARC, sarcoma.

To determine the prognostic value of ENO1 in cancer patients, the correlations of ENO1 expression with prognosis and tumor stage in different cancers were investigated (**Figure 3**). The expression of ENO1 was significantly associated with the prognosis of eight types of cancers, including HNSC (HR = 1.32, *P* = 0.04), CESC (HR = 1.47, *P* = 0.04), BLCA (HR = 1.23, *P* = 0.04), LUAD (HR = 1.36, *P* = 0.01), SARC (HR = 1.36, *P* = 0.00), PAAD (HR = 1.65, *P* = 0.00), KICH (HR = 4.60, *P* = 0.00), and LIHC (HR = 1.63, *P* = 0.00), suggesting that high ENO1 expression might be an independent risk factor for these cancers (all HR > 1.00, *P* < 0.05). Then the survival curves were constructed to further evaluate the prognostic potential of ENO1 (**Figure 4**). High ENO1 expression was significantly associated with worse OS in CESC (log-rank *P* = 0.04), BLCA (log-rank *P* = 0.03), KICH (log-rank *P* = 0.01), LIHC (log-rank *P* = 0.00), and SARC (log-rank *P* = 0.04), and worse DFS in KICH (log-rank *P* = 0.03) and SARC (log-rank *P* = 0.07). Obviously, the expression level of ENO1 was significantly different in the different tumor stages of CESC, LUAD, PICH, PAAD, and LIHC, but not in other types. Correlation of ENO1 expression with prognostic values in all 23 types of cancer is summarized in **Supplementary Table 2**.

**Figure 5:**
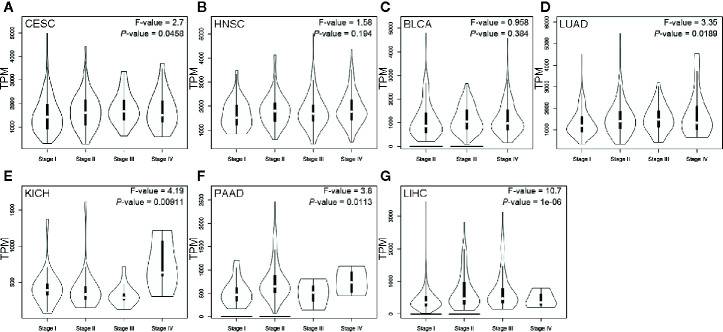
ENO1 expression at different pathological stages of CESC **(A)**, HNSC **(B)**, BLCA **(C)**, LUAD **(D)**, KICH **(E)**, PAAD **(F)** and LIHC **(G)**. Data were analyzed using one-way ANOVA. The ENO1 expression levels are shown in transcripts per million in Y-axes. CESC, cervical squamous cell carcinoma and endocervical adenocarcinoma; HNSC, head and neck squamous cell carcinoma; BLCA, bladder urothelial carcinoma; LUAD, lung adenocarcinoma; KICH, kidney chromophobe; PAAD, pancreatic adenocarcinoma; LIHC, liver hepatocellular carcinoma.

The violin plots were generated to demonstrate the impact of ENO1 expression on tumor stage in these cancers. The data of SARC were not plotted due to small sample size (**Figure 5**). The expression of ENO1 at different pathological stages was significantly different in CESC (*F* = 2.7, *P* = 0.0458), LUAD (*F* = 3.35, *P* = 0.0189), KICH (*F* = 4.19, *P* = 0.00911), PAAD (*F* = 3.8, *P* = 0.0113), and LIHC (*F* = 10.7, *P* = 1e-06), indicating that ENO1 might play a key role in the progression of these cancers.

### Mutation Analysis of ENO1

**Figure 6:**
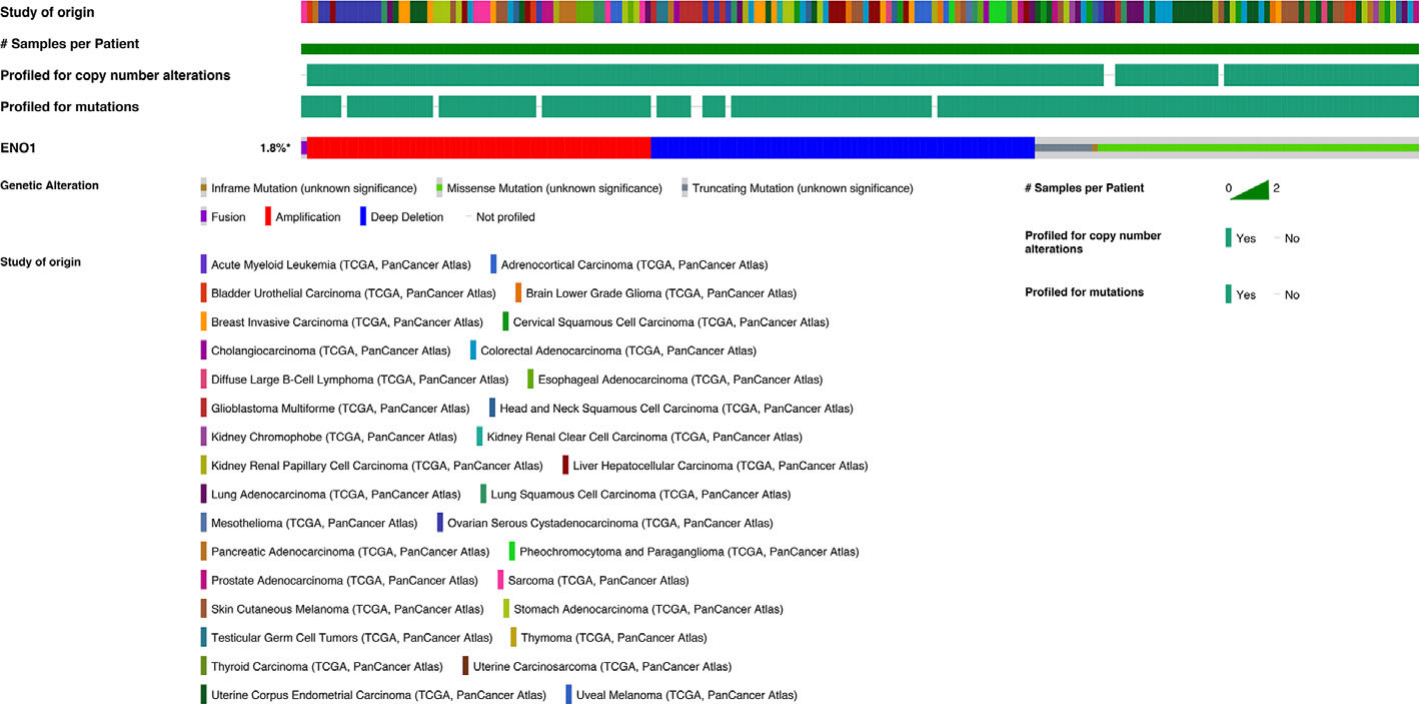
Chart plots of genetic alteration of ENO1 in The Cancer Genome Atlas patients. A total of 1.8% ENO1 was altered including inframe mutation, missense mutation, truncating mutation, fusion, amplification and deep deletion. The detailed information is shown.

**Figure 7:**
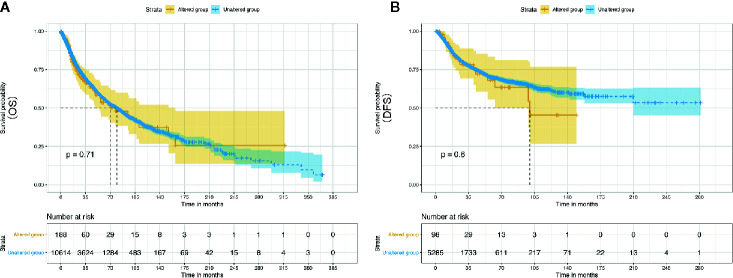
Kaplan–Meier survival curves of the prognostic significance of altered and unaltered of ENO1 using the cBio Portal. Survival curves comparing survival outcomes of patients with altered and unaltered of ENO1 for OS **(A)** and DFS **(B)** with 95% confidence interval.

Evidently, 195 samples in the altered group and 10,772 samples in the unaltered group were included for ENO1 mutation analysis. The results demonstrated that ENO1 was altered in 1.8% of all the included samples, including inframe mutation, missense mutation, truncating mutation, fusion, amplification, and deep deletion (**Figure 6**). Furthermore, the prognostic significance of ENO1 mutation was estimated *via* Kaplan–Meier method. The survival curves indicate that no statistical significance was found between the altered group and the unaltered group either in OS (altered group = 188, unaltered group = 10,614, log-rank *P* = 0.71, **Figure 7A**) or DFS (altered group = 98, unaltered group = 5,285, log-rank *P* = 0.60, **Figure 7B**).

### Correlation Between ENO1 Expression and Immune Infiltrates

**Figure 8:**
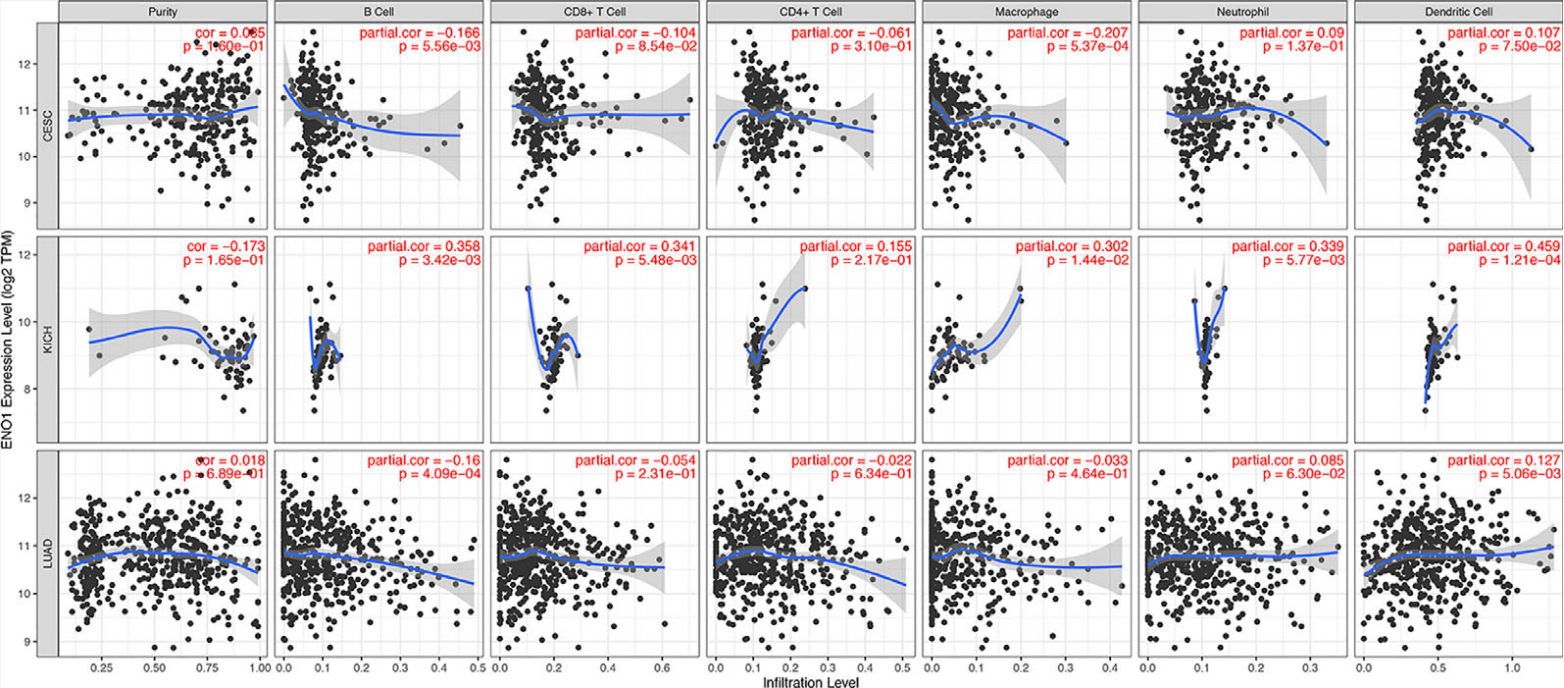
Correlation between ENO1 expression and immune infiltration levels in CESC, KICH, and LUAD. The values of ENO1 expression are shown as log_2_ (transcripts per million). ENO1: enolase 1; CESC, cervical squamous cell carcinoma and endocervical adenocarcinoma; KICH, kidney chromophobe; LUAD, lung adenocarcinoma.

The level of tumor-infiltrating lymphocytes is an independent predictor of sentinel lymph node status and survival outcomes in cancers. We further investigated the correlation between ENO1 expression and the abundance of immune infiltrates (**Figure 8**). In CESC, ENO1 expression was negatively and significantly correlated with the infiltrating levels of B cells and macrophages (all *P* < 0.05). However, no significant correlation was observed between ENO1 expression and tumor purity, as well as the levels of dendritic cells, neutrophils, CD4^+^ T cells, or CD8^+^ T cells (all *P* > 0.05). In KICH, ENO1 expression was positively and significantly correlated to the infiltration of dendritic cells, neutrophils, CD8^+^ T cells, B cells, and macrophages (all *P* < 0.05), but no significant correlation was found with tumor purity or the infiltrating level of CD4^+^ T cells (all *P* > 0.05). In LUAD, the expression level of ENO1 was negatively correlated to the infiltration of B cells but positively associated with the level of dendritic cells (all *P* < 0.05). No significant correlation was found between ENO1 expression and the infiltration of other cells (all *P* > 0.05).

To further explore the potential relationships between ENO1 and infiltrating immune cells, we examined the correlations between ENO1 and several immune cell markers in TIMER and GEPIA. Specifically, we assessed the correlation between ENO1 expression and levels of markers for particular cell subsets including CD8^+^ T cells, B cells, monocytes and other cells. As shown in **Supplementary Table 3**, we adjusted these results based on tumor purity, revealing a significant correlation between ENO1 expression and monocyte markers (CD86, CD115), TAM markers (CCL2, IL10), M1 macrophage markers (INOS, IRF5, COX2), M2 macrophage markers (CD163, VSIG4, MS4A4A), neutrophils markers (CD11b, CD66b), NK cell markers (KIR2DL4), DC markers (BCDA‐A, BDCA‐4, CD11C), Th1 markers (STAT4), Th2 markers (GATA3, STAT5A), Tfh markers (BCL6), Th17 markers (STAT3) and Treg markers (CCR8, STAT5B, TGF*β*1).In LIHC, B cells and T cells were two immune cell types most strongly correlated with ENO1 expression. In KICH, tumor purity had no effect on the relationship between ENO1 and tumor markers. At the same time, T cells, B cells, monocytes and dendritic cells also play an important role in immune infiltration markers in LUAD. In TIMER, after adjustments for tumor purity, the ENO1 expression level was significantly correlated with 14 out of 57 immune cell markers in CESC, 30 out of 57 immune cell markers in KICH, and 24 out of 57 immune cell markers in LUAD. Hence, these results confirm our speculation that ENO1 expression in KICH; CESC and LUAD correlate with immune cell infiltration in different manners, which can help explain the differences in patient survival.

### Kyoto Encyclopedia of Genes and Genomes Pathway Analysis of ENO1

**Figure 9:**
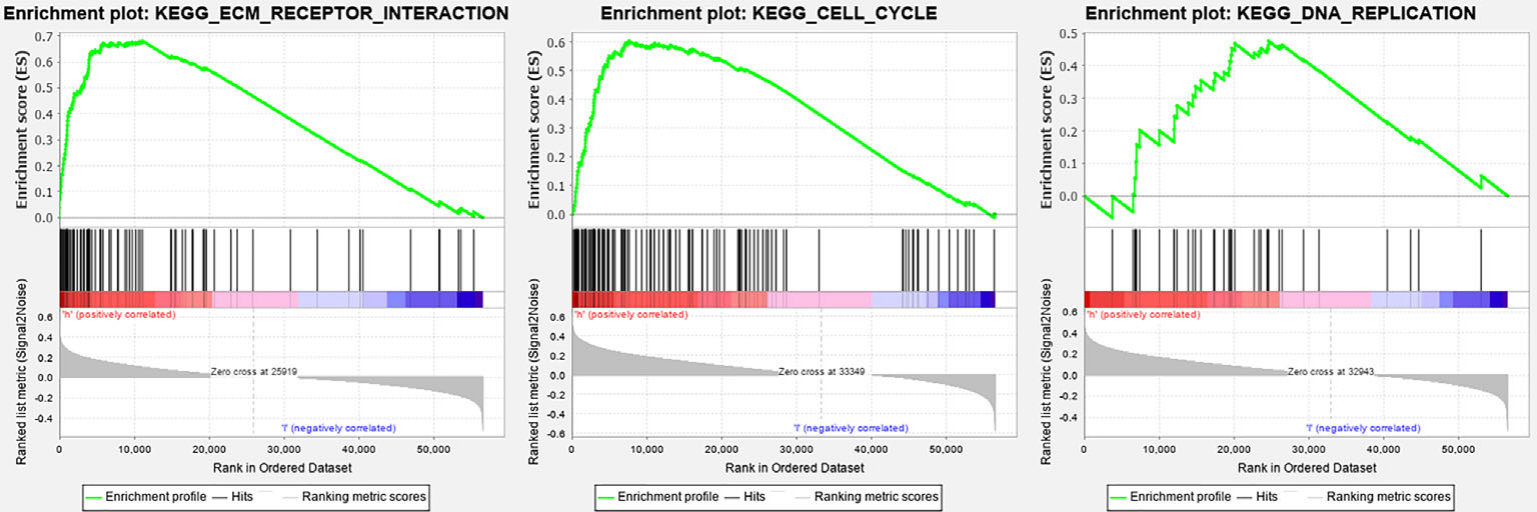
Gene Set Enrichment Analysis of ENO1 in KEGG pathways. The enrichment score increased with the number of enriched genes and *vice versa*. The plots show the curves of ECM receptor interaction (*P* = 0.00), cell cycle (*P* = 0.04), and DNA duplication (*P* = 0.01).

The enrichment score (ES) was calculated using GSEA. The positive ES and negative ES indicated that the gene set was enriched at the top or bottom of the ranked list, respectively. The results revealed that ENO1 was mainly enriched in cell cycle, extracellular matrix (ECM) receptor interaction, DNA replication, apoptosis, and glycolysis process (**Figure 9**).

## Discussion

In the present study, the correlations of ENO1 with prognosis, tumor stage, and immune infiltrating levels in multiple cancers were investigated *via* analyzing TCGA data. ENO1 is a bifunctional gene that encodes a glycolytic protein and a c-Myc-binding protein (39). The involvement of ENO1 in a variety of pathways, particularly glycolysis-related pathways, is closely related to tumor formation and progress. Previous studies have reported the effects of ENO1 on tumor development (15, 17–19, 40–45), which support our results showing that ENO1 is significantly correlated with prognosis, tumor stage, and immune infiltrates in cancers.

Tumor-infiltrating immune cells can be effectively targeted by anti-cancer agents and the subtle alterations in their composition and function are correlated with the clinical outcomes of cancer patients (18, 46). In recent years, there has been growing interest in understanding the role of immune system in the initiation and progression of cancers (47). Clinical evidence has suggested the effectiveness of immunotherapy for subsets of patients with advanced tumors (48). Cancer immunotherapy is mostly based on the upregulation of tumor antigens (49). ENO1 has been found to induce autoantibody production in patients with cholangiocarcinoma, breast tumor, head and neck tumor, leukemia, lung tumor, pancreatic tumor, and melanoma (26, 28, 50–52). ENO1-specific T cells can recirculate from the tumor to the periphery despite different functional profiles. The presence of peripheral ENO1-specific T cells is significantly correlated with improved survival, suggesting the prognostic value of these cells in cancers (17). The correspondence between peripheral and intratumoral ENO1-specific immune responses has been demonstrated, and the circulating ENO1-specific T cells exhibited an effective anticancer effect in pancreatic ductal adenocarcinoma (17). In addition, the enzymatic activity of ENO1 in solid tumors is regulated at posttranslational level as evidenced by the upregulated mRNA and protein expressions of ENO1 in CD8^+^ tumor-infiltrating lymphocytes (45). Consistently, our results also showed a significant correlation between ENO1 expression and immune infiltrates. Interestingly, tumor infiltration has no significant effect on the cumulative survival (**Supplementary Figure 1**).

The KEGG analysis in this study revealed that ENO1 was mainly enriched in DNA replication, cell cycle, apoptosis, glycolysis process, and ECM receptor interaction. These findings are consistent with a previous study showing that high ENO1 expression is significantly correlated with DNA replication and cell cycle in hepatocellular carcinoma (19). One of the roles of ENO1 is to act as a plasminogen receptor linked to increased cellular inflammation, migration, and invasion *via* ECM remodeling (53), suggesting that the downregulation of ENO1 on cell surface may suppress other tumorigenic processes (41). ENO1 also shows a regulatory effect on cell cycle. Cancer cells are characterized by aberrant cell cycle activity and unlimited replicative potential. The therapies targeting cell-cycle proteins have been used for the treatment of multiple cancers, including prostate cancer, breast cancer, and lung carcinoma (54). ENO1 is an oncogene that promotes cell cycle progression, proliferation, migration, and invasion. The overexpression of ENO1 increased the levels of oncogenic cell cycle regulators in non-small cell lung cancer (18). ENO1 also regulated apoptosis and cell cycle in bladder and pancreatic cancer cells (42, 43). Furthermore, the knockdown of ENO1 has been shown to promote apoptosis and induce the arrest of cell cycle in gastric cancer cells (15). According to previous findings, circ-ENO1 and its host gene ENO1 were upregulated in LUAD cells (2). ENO1 might also contribute to the progression of lung cancers by stimulating cell proliferation *via* accelerating G1/S transition, but not in esophageal cancers (44). Taken together, the varied expression of ENO1 in different types and stages of cancers implied that the effects of ENO1 may vary in different cancers and at different tumor stages. However, the potential mechanisms involved in the regulation of ENO1 in cancers needs to be further explored. Additionally, mutation of ENO1 was analysis, for it was proved that mutation could affect tumor progression (55, 56); however, ENO1 mutation (1.8%) did not impact on prognosis in this study.

The Tumor-Node-Metastasis (TNM) classification aims to improve the management of cancers including cancer control, research design, clinical care guidance and decision making (57). As demonstrated in another study, TNM stage played a critical role in survival in metachronous lung cancer (58). To build up a comprehensive prognosis predicting and strategies determining system, all relevant factors should be considered, including TNM stage. In this study, differential expression of ENO1 in different stages of CESC, HNSC, BLCA, LUAD, KICH, PAAD, and LIHC was identified, indicating ENO1 could be a marker for tumor staging. In consonance with our results, ENO1 expression was higher in late stages (stages III and IV), particularly that in KICH (**Figure 5E**), meanwhile, high ENO1 expression was significantly associated with worse OS in KICH (**Figure 4I**). Further to our previous statement that ENO1 could promote cancer progression *via* stimulating cell proliferation, increasing invasion and migration, and other mechanisms, ENO1 may lead to a late stage on the aspect of metastasis. There is still uncertainty, however, whether ENO1 is a determinant of tumor stage.

Our results showed that ENO1 expression was significantly higher in normal tissues as compared to KICH tissues, whereas high expression of ENO1 predicted poor OS in other cancers. Notably, the downregulation of ENO1 has been found in tissue samples from patients with non-small cell lung cancer, and the patients with low ENO1 expression had a worse prognosis (40). These results indicate a contradictory role of ENO1 in tumor formation. KICH is a rare carcinoma originating from the collecting duct and is typically the least aggressive subtype of renal cell carcinomas with a good prognosis unless characterized by sarcomatous transformation (59, 60). Although small number of deaths and advanced cases may lead to bias in the analysis of ENO1 expression and survival outcomes, the potential biological function of ENO1 and its correlation to survival are worth further investigation.

There are some limitations in the current study. First, the treatments given to patients might affect the expression of ENO1, leading to a potential bias. Additionally, patients with advanced cancers were underrepresented in TCGA cohort, particularly the ones with KICH, while ENO1 might show different biological activities at different tumor stages. Efforts should be directed towards the preparation of prospective clinical trials to evaluate the prognostic value of ENO1 as a tumor marker in cancers at different stages.

ENO1 significantly regulates macrophage infiltration in CESC and LUAD. In addition, patients with CESC and LUAD had poor clinical outcomes and high macrophage infiltration. Taken together, these analyses reveal the clinical importance of ENO1 as a macrophage infiltration regulator in patients with CESC and LUAD. These results reveal the potential regulatory role of ENO1 in tumor associated macrophage polarization. In summary, this pan-cancer analysis demonstrated that increased ENO1 expression was correlated with poor prognosis and decreased immune infiltration levels in macrophages, CD4^+^ T cells, CD8^+^ T cells, and B cells in CESC and LUAD. However, the opposite effect of ENO1 on immune infiltration was observed in KICH. The expression of ENO1 also potentially contributed to the stages of tumor development. Therefore, ENO1 may be used as a potential biomarker for predicting prognosis, tumor stage, and immune infiltration in CESC, LUAD, and KICH patients.

## Data Availability Statement

The datasets presented in this study can be found in online repositories. The names of the repository/repositories and accession number(s) can be found in the article/**Supplementary Material**.

## Author Contributions

Conceptualization: JZ, HL, and WX. Methodology: HL, WX, WY, and CW. Investigation: WX, WY, and CW. Visualization: WX, WY, and CW. Manuscript draft: HL, WX, and CW. Manuscript review and editing: HL, WX, WY, CW, XM, and JZ. Supervision: HL and JZ. All authors contributed to the article and approved the submitted version.

## Conflict of Interest

The authors declare that the research was conducted in the absence of any commercial or financial relationships that could be construed as a potential conflict of interest.

## Abbreviations

ENO1, enolase 1; TCGA, The Cancer Genome Atlas; OS, overall survival; BLCA, bladder urothelial carcinoma; BRCA, breast invasive carcinoma; CESC, cervical squamous cell carcinoma and endocervical adenocarcinoma; CHOL, cholangiocarcinoma; COAD, colon adenocarcinoma; ESCA, esophageal carcinoma; HNSC, head and neck squamous cell carcinoma; KICH, kidney chromophobe; KIRC, kidney renal clear cell carcinoma; KIRP, kidney renal papillary cell carcinoma; LIHC, liver hepatocellular carcinoma; LUAD, lung adenocarcinoma; LUSC, lung squamous cell carcinoma; PAAD, pancreatic adenocarcinoma; PCPG, pheochromocytoma and paraganglioma; PRAD, prostate adenocarcinoma; READ, rectum adenocarcinoma; SKCM, skin cutaneous melanoma; STAD, stomach adenocarcinoma; THCA, thyroid carcinoma; THYM, thymoma; UCEC, uterine corpus endometrial carcinoma; GEPIA, Gene Expression Profiling Interactive Analysis; DFS, disease free survival; GSEA, Gene Set Enrichment Analysis; TIMER, Tumor Immune Estimation Resource; TPM, transcripts per million; HR, hazard ratio; KEGG, Kyoto Encyclopedia of Genes and Genomes; ES, enrichment score.
