# Supplementary material for: Enolase 1 Correlated With Cancer Progression and Immune-Infiltrating in Multiple Cancer Types: A Pan-Cancer Analysis
Source: Front Oncol. 2021 Feb 10;10:593706. doi: 10.3389/fonc.2020.593706 (PMC7902799; doi:10.3389/fonc.2020.593706)
Supplement: Supplementary file 1 [file Table_1.docx]

**Supplementary Table 1 Correlation of ENO1 expression with prognostic significance in multiple types of cancer in the GEPIA (OS and DFS).**

| Cancers | OS | | DFS | |
| --- | --- | --- | --- | --- |
|  | HR (high expression) | *P* (log-rank) | HR (high expression) | *P* (log-rank) |
| BLCA | 1.40 | 0.0250 | 1.30 | 0.0950 |
| BRCA | 1.20 | 0.2800 | 1.10 | 0.5100 |
| CESC | 1.60 | 0.0440 | 0.93 | 0.8100 |
| CHOL | 0.72 | 0.5000 | 0.67 | 0.3800 |
| COAD | 0.87 | 0.5700 | 0.83 | 0.4600 |
| ESCA | 0.97 | 0.9100 | 0.99 | 0.9700 |
| HNSC | 1.10 | 0.3100 | 1.20 | 0.2100 |
| KICH | 10.00 | 0.0076 | 4.90 | 0.0270 |
| KIRC | 0.61 | 0.0014 | 0.75 | 0.1100 |
| KIRP | 0.79 | 0.4500 | 0.51 | 0.0240 |
| LIHC | 1.90 | 0.0005 | 1.30 | 0.0870 |
| LUAD | 1.30 | 0.0620 | 1.30 | 0.1000 |
| LUSC | 0.97 | 0.8400 | 1.40 | 0.0420 |
| PAAD | 1.10 | 0.5400 | 1.50 | 0.0570 |
| PCPG | 0.52 | 0.4500 | 1.60 | 0.3400 |
| PRAD | 1.60 | 0.4600 | 1.30 | 0.2000 |
| READ | 1.10 | 0.8200 | 0.88 | 0.7900 |
| SARC | 1.50 | 0.0420 | 1.60 | 0.0068 |
| SKCM | 1.10 | 0.5500 | 0.93 | 0.5600 |
| STAD | 0.83 | 0.2400 | 1.00 | 0.9000 |
| THCA | 1.20 | 0.7400 | 0.93 | 0.8100 |
| THYM | 0.71 | 0.6300 | 0.88 | 0.7800 |
| UCEC | 0.87 | 0.7000 | 1.20 | 0.6300 |

ENO1: enolase 1; GEPIA: Gene Expression Profiling Interactive Analysis; HR: hazard ratio; OS: overall survival; DFS: disease free survival.
