# Supplementary material for: Enolase 1 Correlated With Cancer Progression and Immune-Infiltrating in Multiple Cancer Types: A Pan-Cancer Analysis
Source: Front Oncol. 2021 Feb 10;10:593706. doi: 10.3389/fonc.2020.593706 (PMC7902799; doi:10.3389/fonc.2020.593706)
Supplement: Supplementary file 2 [file Table_2.docx]

**Supplementary Table 2 Expression and biological function of ENO1 in pan-cancer.**

| Type | Research | Year | Samples | Expression | Biological function |
| --- | --- | --- | --- | --- | --- |
|  |  |  | (N/T) | of ENO1 |  |
| GC | Xu (14) | 2019 | 34/125 | Upregulated | Promoting epithelial-mesenchymal transition of GC cells. |
| GC | Qiao (15) | 2019 | 228/410 | Upregulated | Anti-apoptosis and promoting proliferation of GC cells. |
| BC | Ji (16) | 2019 | 30/414 | Upregulated | Regulating cell cycle and apoptosis of BC cells. |
| PDAC | Niccolai (17) | 2016 | 15/15 | Upregulated | ENO1-specific T cells has a prognostic value and significantly correlates with a longer survival. |
| NSCLC | Fu (18) | 2015 | 36/36 | Upregulated | Promoting glycolysis, growth, migration, and invasion of NSCLC. |
| HNC | Tsai (4) | 2010 | 44/44 | Upregulated | ENO1-mediated promotion of cell transformation and invasion partly via induced CCL20 expression. |
| LUAD | Zhou (2) | 2019 | 64/64 | Upregulated | Circ-ENO1 promoted glycolysis and tumor progression in LUAD by miR-22-3p/ENO1 axis. |
| HCC | Zhu (19) | 2018 | 50/374 | Upregulated | NA |
| PC | Yin (20) | 2018 | 50/73 | Upregulated | NA |
| HCC | Luo (21) | 2019 | 18/104 | Upregulated | NA |
| NSCLC | Yu (22) | 2014 | 64/64 | Upregulated | NA |
| KICH | White (23) | 2015 | 360/360 | Downregulated | NA |

Notes: N, normal group; C, cancer group; Expression of ENO1, the expression level of ENO1 in the cancer group compared with the control group; NA, not applicable.
