# Supplementary figures and images for: Enolase 1 Correlated With Cancer Progression and Immune-Infiltrating in Multiple Cancer Types: A Pan-Cancer Analysis
Source: Front Oncol. 2021 Feb 10;10:593706. doi: 10.3389/fonc.2020.593706 (PMC7902799; doi:10.3389/fonc.2020.593706)

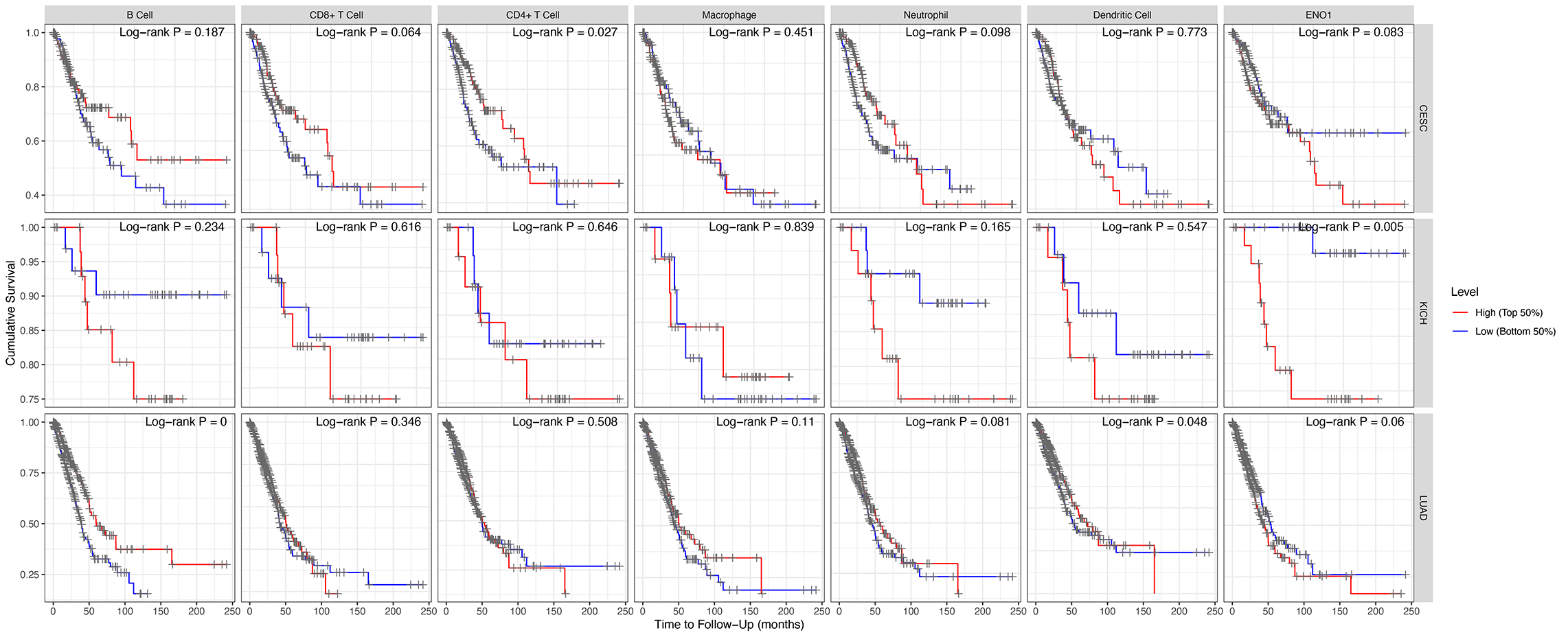

Supplement: Supplementary file 4 [file Image_1.tif]
